# Importance of Client Orientation Domains in Non-Clinical Quality of Care: A Household Survey in High and Low Income Districts of Mashhad

**DOI:** 10.5539/gjhs.v8n7p228

**Published:** 2015-12-16

**Authors:** Somayeh Fazaeli, Mehdi Yousefi, Seyed Hasan Banikazemi, Seyed Amir Hossein Ghazizadeh Hashemi, Ali Khorsand Vakilzadeh, Narges Hoseinzadeh Aval

**Affiliations:** 1 School of Paramedical Sciences, Mashhad University of Medical Sciences, Mashhad, Iran; 2 School of Health, Mashhad University of Medical Sciences, Mashhad, Iran; 3 Health Strategic Research Center, School of Health, Mashhad University of Medical Sciences, Mashhad, Iran; 4 School of Persian and Complementary Medicine, Mashhad University of Medical Sciences, Mashhad, Iran; 5 Sheikh Hospital, Mashhad University of Medical Sciences, Mashhad, Iran

**Keywords:** responsiveness, quality of care, client orientation, households

## Abstract

Responsiveness introduced by WHO as a key indicator to assess the performance of health systems and measures by common set of domains that are categorized in to two main categories “Respect for persons” and “client orientation”. This study measured importance of client orientation domains in high and low income districts of Mashhad. In this cross-sectional and explanatory study, Sample of 923 households were selected randomly from two high and low income districts of Mashhad. World Health Organization (WHO) questionnaire was used for data collection. Standard frequency analyses and Ordinal logistic regression (OLR) was employed for data analysis. In general, respondents selected quality of basic amenities as the most important domain and access to social support networks was identified as the least important domain. Households in high income area scored higher domains of prompt attentions and choice Compared to low income. There was a significant relationship between variables of ages, having member that need to care and self-assessed health with the ranking of client orientation domains.

Study of households’ view on ranking of non-clinical aspects of quality of care, especially when faced with limited resources, can help to conduct efforts towards subjects that are more important, and lead to improve the health system performance and productivity.

## 1. Introduction

WHO identified responsiveness as one of the key goals to which health systems contribute in improving population health and for facilitating of its measurement in a systematic way across countries, developed a common set of domains (Hsu, Chen, Hu, Yip, & Shu, 2006; Murray & Frenk, 2000), that were categorized in to two main categories (Campbell, Roland, & Buetow, 2000): “Respect for persons” and client orientation”. Respect for persons refer to intending to capture ethical aspects of the interaction between individuals and the health system, includes three sub-elements: dignity, autonomy and confidentiality (Campbell et al., 2000; Fazaeli Ahmadi, Rashidian, & Sadoughi, 2014). And client orientation gauges the components of consumer satisfaction includes four sub-elements: prompt attention, quality of basic amenities, access to social supports networks (during inpatient care) and choice of care providers (Hsu et al., 2006; Valentine, Darby, & Bonsel, 2008).

WHO Claimed these domains have ‘‘universal’’ importance, it means that are important for all humans, regardless of culture, sex, age and so on. Of course, WHO expressed a serious concern about exploring users’ priorities with respect to different aspects of health services (Valentine et al., 2008).

Some studies showed that usually there are divergences in priorities ‘‘between individual patients and between patients from different cultures and health care systems and individual characteristics such as education, health status, sex and age (Jung, Baerveldt, Olesen, Grol, & Wensing, 2003; Wensing, Jung, Mainz, Olesen, & Grol, 1998), also some studies have reported weak associations between priorities and individual (or household) socio-economic characteristics (Fung et al., 2005; Rashidian et al., 2011; Sofaer, Crofton, Goldstein, Hoy, & Crabb, 2005). This difference may lead to conflicts and sometimes even lack of satisfaction (Valentine et al., 2008). Therefore, determining the relative importance of non-clinical aspects of quality of care among various subgroups (base on income, culture and etc.), can be useful in providing a correct interpretation of health services users’ needs and help to optimize the allocation of health system resources (Malhotra & Do, 2012; Wensing & Elwyn, 2003; M. Yousefi, Assari Arani, Sahabi, Kazemnejad, & Fazaeli, 2014). Despite of importance of this subject, we have few studies (especially in Iran) in this area compared to other health system subjects (Rashidian et al., 2011; Valentine et al., 2008) and the previous studies have generally done on the concept of measuring patient satisfaction (Aghajani, 2007; Cronholm & Goldkuhl, 2003; Siamian Gonbadi, Nasiri, & Shahrabi, 2005).

The main objective of this study was to determine the relative importance of sub-elements related to domain of client orientation of non-clinical aspects of quality of care “responsiveness” among selected districts of Mashhad.

## 2. Materials and Methods

This cross-sectional and explanatory study was performed in 2014. Households that were resident in low and high income district of Mashhad, were the statistical population of study (Yousefi, 2010). Sample size for each district was calculated through Cochran Sample Size Formula (p=0.5 for maximum variability, 95% confidence level and ±5% precision). Finally, sample size was 500 households in every district (totally 1000 households).

Multi stage sampling was used for sample selection. After determination of classes, each class divided to clusters with similar characteristics (city blocks), each block was the area bounded by four streets. Then the researcher specified the number of samples of each cluster on a regular basis among households in selected districts.

The used instrument in this study, was the WHO questionnaire (included responsiveness module contains questions about “importance of responsiveness domains from people’s view” and demographic characteristics of households). Questionnaire was translated to Persian and confirmed its validity and reliability in study of Rashidian et al. (2011). Table 1 provides brief descriptions of client orientation elements in questionnaire (Valentine et al., 2008).

**Table 1:** descriptions of sub-elements of client orientation

Sub Elements of client orientation	Brief Description
Prompt Attention	•having a reasonable distance and travel time from your home to the health care provider •getting fast care in emergencies •short waiting times for appointments and consultations, and getting tests done quickly •short waiting lists for non-emergency surgery
Choice	•being able to choose your doctor or nurse or other person usually providing your health care •being able to go to another place for health care if you want to
Quality Of Basic Amenities	•having enough space, seating and fresh air in the waiting room •having a clean facility (including clean toilets) •having healthy and edible food
Social Support	•being allowed the provision of food and other gifts by relatives while in hospital •being allowed freedom of religious practices

An eligible respondents (18 years or older, preferably parents) were selected as participant. Questioners were trained before the start of data collection in this study about study subject, questions, maintenance of confidentiality of household’s information, sampling methods and interviewing method. Accordingly, in the first contact, the questioner has given some information to participant based on the study guide (included description of the study aims, sponsor and questions and etc.). Completion of questioner took between 15 to 25 minutes. All participants were requested to sign or mark (if illiterate) an informed consent form. If a household did not tend to participate in the study or was not present at home after three times referring, based on the sample selection guideline, a new household was replaced. The five-point Likert scale (extremely important, very important, important, fairly important, and not at all important from 5 to 1) was applied. Also this study was approved by the Ethics Committee of the Mashhad University of Medical Sciences. Standard frequency analyses were reported for each importance question by district and for socio demographically Characterizes of households include: sex, age, education, health status (self-reported health). Ordinal logistic regression (OLR) was employed to assess the role of the ten variables on the households’ view on the importance of client orientation’s sub elements. All analyses were performed using SPSS 19.

## 3. Results

A total of 480 householders in low and 443 householders in high income districts completed the questionnaires. Examination of demographic data of participants showed that there was at least one person under the age of 12 in about 41 % of households. More than 62% of them reported their health conditions as good and very good. Extra demographic data has been presented in Table 2.

**Table 2:** Percentage of respondents selecting sub elements as very important

demographic characteristics of the study sample		prompt attention		choice		quality of basic amenities		social support	

		n %	z(sig.)	n %	z(sig.)	n %	z(sig.)	n %	z(sig.)
**districts: low/high income**	low(n=480)	47.6%	-0.736 (0.462)	41.4%	-2.792 (0.005) **	60.4%	-0.872 (0.383)	31.6%	-2.741 (0.006) **
			
	high(n=443)	49.4%		50.5%		58.5%		26.4%	

**Sex**	male (n=448)	48.0%	-0.803 (0.422)	47.6%	-0.876 (0.381)	56.5%	-1.805 (0.071)	29.3%	-0.201 (0.841)
			
	female(n=441)	49.5%		44.3%		62.6%		29.1%	

**<12 years member living in household**	yes(n=383)	47.4%	-0.079 (0.937)	45.0%	-0.820 (0.412)	60.4%	-1.130 (0.258)	29.0%	-0.680 (0.497)
			
	no(n=535)	49.1%		46.1%		58.9%		29.4%	

**self-assessed health**	good and very good(n=559)	47.6%	0.455 (0.797)	45.0U	0.171 (0.918)	59.9%	0.311 (0.856)	30.9%	9.958 (0.007) **
			
	moderate(n=285)	50.7%		46.3%		59.2%		25.0%	
			
	bad and very bad (n=53)	48.1%		53.8%		58.0%		32.7%	

**65+years member living in household**	yes(n=262)	50.6%	-0.629 (0.529)	48.9%	-1.496 (0.135)	56.7%	-1.258 (0.208)	28.7%	-0.441 (0.659)
			
	no(n=648)	47.5%		44.3%		60.5%		29.0%	

**member with needed care living in household**	yes(n=252)	58.1%	-2.724 (0.006) **	51.4%	-1.516 (0.130)	59.4%	-0.053 (0.957)	33.6%	-1.918 (0.055) *
			
	no(n=656)	45.3%		43.8%		59.5%		27.7%	

**using of health services in the past year /more than one year ago**	during past year(n=716)	50.7%	-2.148 (0.032) *	46.8%	-1.188 (0.235)	59.7%	-0.256 (0.798)	29.8%	-2.322 (0.020) *
			
	more than one year ago(n=179)	41.5%		42.4%		59.9%		26.6%	

**Insurance**	have(n=558)	50.5%	-0.285 (0.775)	45.0%	-2.053 (0.040) *	60.7%	-0.103 (0.918)	30.8%	-1.317 (0.188)
			
	don’t have(n=289)	49.8%		49.8%		61.1%		25.9%	

**literacy (years)**	0-6(n=80)	42.9%	1.908 (0.385)	35.9%	3.887 (0.143)	46.2%	8.301 (0.016)*	34.6%	19.197 (0.000)**
			
	6-11(n=494)	48.3%		44.9%		60.8%		33.2%	
			
	12 <(n=323)	51.9%		50.2%		63.1%		22.4%	

** Correlation is significant at the 0.01 level (2-tailed).

* Correlation is significant at the 0.05 level (2-tailed).

The findings shows that participants identified the quality of basic amenities as the most important sub-element among different client orientation’s sub-elements and after that prompt attention, choice and social support have the most importance respectively.

From the Table 1 we can see that a significant relation between districts, literacy and self-assessed health with importance of social support (P-Value≤ 0.01). In addition, there was a statistically significant difference between important scores in social support and using of health services or having member that needs care in household (P-Value ≤ 0.05).

Also Significant differences were found in ranking prompt attention in terms using of health services in the past year /more than one year ago(P-Value ≤ 0.05) and having member that needs care in household (P-Value ≤ 0.01). As table 1 show, quality of amenities is significantly related to literacy of responders (P-Value ≤ 0.01). Also score of importance of choice of provider was significantly difference between two districts (P-Value ≤ 0.01) and also between households with and without insurance (P-Value ≤ 0.05).

Table 3 shows which demographics factors effect on the selecting client orientation sub-elements as very important.

**Table 3:** Determinants of selecting client orientation as very important [with 95% confidence intervals] from the ordinal logistic regression (OLR)

Variables	B	S.E.	Wald	Sig.	95% Confidence Interval	

					Lower Bound	Upper Bound
age (years)	0.009	0.004	6.036	0.014	0.002	0.016
Self-assessed health (very good)	-0.172	0.067	6.523	0.011	-0.304	-0.040
Education (year)	0.014	0.013	1.213	0.271	-0.011	0.040
Be in Higher levels of income	0.00	0.00	1.56	0.21	0.00	0.00
Higher household size	-0.036	0.031	1.302	0.254	-0.097	0.026
High income district Settlement	-0.003	0.237	0.000	0.991	-0.467	0.462
Female responder	-0.122	0.101	1.461	0.227	-0.320	0.076
65>years member living in household	0.240	0.114	4.425	0.035	0.016	0.464
12 <years member living in household	0.047	0.114	0.171	0.679	-0.176	0.270
member with needed care in household	-0.280	0.117	5.688	0.017	-0.509	-0.050

Model statistics: LR χ^2^=23.47 (P. value=0.015), Pseudo R-Square=0.044. (Link function: Complementary Log-log.)

Logistic regression analyses showed that Age has a positive effect on level of importance of client orientation elements. Self-assessed health (mentioned as very good) of responders and having member that need to care in household have a negative effect on the level of importance of client orientation sub-elements that is mentioned by households.

## 4. Discussion

Responsiveness expresses respect for human rights in the health care systems and Measures the level of fulfillment of legitimated expectations of people from health system (Karami-Tanha, Moradi-Lakeh, Fallah-Abadi, & Nojomi, 2014).

Responsiveness has two main areas that this study determines the relative importance of each element associated with the client orientation from the perspective of the households in high and low income districts. Ranking these areas from the perspective of people with different economic, social and cultural characteristics has been emphasized in several studies (Valentine et al., 2008).

Generally, the results from this study showed that quality of basic amenities has been selected as the most important element from the perspective of participants as well as the studies conducted by Rashidian et al in the district 17 of Tehran, Karami et al among heart inpatients in hospital and kowal et al in Asia (Kowal, Naidoo, Williams, & Chatterji, 2011; Rashidian et al., 2011).

The similarity of these results does not mean the same expectations, but these results shows quality of basic amenities has been the most important element compared to other elements of client orientation. But considering the scale of measurement of importance level of the sub-elements of client orientation, the importance of these areas cannot be proved in various studies.

The next point in the current study is the similarity of this priority between high and low income districts that shows that even households who live in districts with low income, also expect to receive services with an appropriate quality of service. This can be paid more attention when we know that the significant part of outpatient health services in Mashhad are similar between households in high income and those in low income districts. But in addition to the priorities set and based on Results from WHO’s general population surveys of “health system responsiveness” in 41 countries, which was reported in 2008, the most important domain for Iranian participants was prompt attention (31%) (Valentine et al., 2008). This result can be seen in some other studies (Karami-Tanha et al., 2014; Liabsuetrakul Petmanee, Sanguanchua, & Oumudee, 2012; Peltzer & Phaswana-Mafuya, 2012; Valentine et al., 2008).

Findings of this study are consistent with previous studies in Iran and is different with studies outside of Iran in setting priorities. This difference may be explained through Valentine’s findings. Valentine explains this difference as follows: “Across subgroups within countries, convergence was stronger than convergence across countries, indicating that health system investments, culture and the human development context were stronger influences on populations’ priorities for their health systems than individual level factors like age, sex, education, health status, and utilization of health services” (Valentine et al., 2008).

In the other hand, the quality of basic amenities not only affects the patient’s comfort, but also is associated with the feeling of promoting health, wellbeing and acceleration in recovery processes (Knaul & Frenk, 2005). However, some studies have shown that there is a gap between patients’ needs and access to basic desired amenities even in developed countries. Undesirability of basic amenities may put the patient at risk (Vafaee-Najar, Pourtaleb, Ebrahimipour, & Dehnavieh, 2013).

Valentine in his study showed that setting priorities in responsiveness domains in field of client orientation is more associated with geographic area as well as the level of human development and in some cases the level of health expenditure. Also in this study it was observed the significant relationship between the type of location and paying attention to the choice right statistically. This means that people living in the low income district has the higher priority for the choice compared to high income district (Valentine et al., 2008).

Some studies have found that older respondents pay attention to autonomy a little more than younger one. However, in this study and the study conducted by Rashidian there was no significant relationship between independency and demographic characteristics of people (Sofaer et al., 2005). Coulter in the study on eight European countries showed most people (51%) preferred the model of joint decision-making and 31 % of people over 55 years, expressed that the doctor should decide (Vafaee-Najar et al., 2013).

In his study, like any other study there were some limitations. The weak willingness of households to participate in these kinds of studies was one of the main limitations this study faced with. To overcome these limitations, we tried to determine the appropriate time by representative from households to complete the questionnaire and used promotional tools and strengthen the communication skills of interviewers as well. Another limitation was the cultural issues during a visit to the home that was resolved by training interviewers and using researchers in both sexes, as well as obtaining the required legal permissions. Also, the low relationship between importance prioritize and individual characteristics may be partly explained by the omission of individual characteristics like ethnicity which was found to be an important determinant in some studies (Fischer, Shumway, & Owen, 2002).

Policy makers in health system can apply these results in prioritizing their efforts when faced with resource constraints (Fazaeli et al., 2014; Kerssens, Groenewegen, Sixma, Boerma, & Eijk, 2004). Because without the understanding of the priorities in the community, efforts to reform and improve health system performance that often focuses on tangible benefits, such as revenues and costs, may be misguided. This may be due to the fact that many of the costs such as the cost caused by the lack of a convenient accessibility of patients to needed services or caused by lack of good quality of basic amenities as an important priority cannot be understood by the usual data in performance assessment. Therefore, the design of appropriate mechanisms that allows prioritizing by people in order to planning to do reform the health system is one of the related fields of policy making in the improvement of health system responsiveness.
